# On patterns and re-use in bioinformatics databases

**DOI:** 10.1093/bioinformatics/btx310

**Published:** 2017-05-19

**Authors:** Michael J Bell, Phillip Lord

**Affiliations:** School of Computing Science, Newcastle University, Newcastle-upon-Tyne, UK

## Abstract

**Motivation:**

As the quantity of data being depositing into biological databases continues to increase, it becomes ever more vital to develop methods that enable us to understand this data and ensure that the knowledge is correct. It is widely-held that data percolates between different databases, which causes particular concerns for data correctness; if this percolation occurs, incorrect data in one database may eventually affect many others while, conversely, corrections in one database may fail to percolate to others. In this paper, we test this widely-held belief by directly looking for sentence reuse both within and between databases. Further, we investigate patterns of how sentences are reused over time. Finally, we consider the limitations of this form of analysis and the implications that this may have for bioinformatics database design.

**Results:**

We show that reuse of annotation is common within many different databases, and that also there is a detectable level of reuse between databases. In addition, we show that there are patterns of reuse that have previously been shown to be associated with percolation errors.

**Availability and implementation:**

Analytical software is available on request.

## 1 Introduction

It is estimated that over 1500 active databases are currently in existence (Fernández-Suárez and Galperin, 2013). While these are generally thought of as containing biological data, they often also contain collected and collated information about the data they carry, which is described as *annotation*. There are many different types of annotation (Wooley and Lin, 2005): some is highly structured and organized containing, for instance, links through to other databases, ontology terms, or taxonomic relationships; others include unstructured or semi-structured free text. The free text, or *textual annotation*, is often considered to be the highest value annotation, although, by its very nature it is also the hardest to represent and analyze computationally (Lord *et al.*, 2001). In this paper, we will consider this form of annotation.

Biological databases and annotation mirror the evolution of biological systems. As highly similar genes occur in many different organisms, transferred both horizontally and vertically, so the annotation about these genes is reused between different databases (Richardson and Watson, 2013). This form of reuse substantially reduces the work required by database annotators, but also creates a problem; for most databases it is difficult to determine the source or support for a particular statement (Bolleman *et al.*, 2010). Although most databases contain out-going references, either to the primary literature or to other databases, these are generally given at the level of the database record. For the richest databases, there may be many statements for each record. In short, databases often lack a formal representation of their *provenance* (Bolleman *et al.*, 2010).

While this form of reuse is part of the folk-history of bioinformatics (http://madhadron.com/posts/2012-03-26-a-farewell-to-bioinformatics.html), and is apparent from even a short perusal of a few bioinformatics databases, it has rarely been explicitly studied. In a previous study (Bell *et al.*, 2013), we have shown that the level of reuse in UniProtKB is extremely high—the most reused sentence in TrEMBL occurs more than seven million times, while the most common sentence in Swiss-Prot occurs more than 91 000 times. Moreover, we have shown that this reuse operates as an informal indicator of provenance; two identical sentences are likely to share a common history. This, in turn, allowed us to identify *propagation patterns* that can be used to detect inconsistencies and errors in this annotation.

Our previous analysis looked at only a single database; but we also believe that reuse occurs between different databases, forming a biological knowledge ecosystem. In this paper, we extend the analysis further, looking at several different databases, and show that *within* these there are also high levels of reuse. The same analysis also allows us to track reuse *between* databases and show that, here also, there is significant reuse. Further, we look for tell-tale signature patterns previously shown to indicate erroneous annotation and show that these patterns are also present within several databases and can be seen between several databases. This analysis suggests that as well as reuse being common-place, that it is possible to detect knowledge flow between databases, giving an informal mechanism for detection of provenance.

## 2 Materials and methods

### 2.1 Choosing a set of databases

There are many databases in the bioinformatics ecosystem that we could use to study. Unfortunately, these vary significantly technologically, both in terms of their format, their identifiers and their scheme for updates and maintenance history. Our previous analysis focused exclusively on UniProtKB, using it an exemplar gold standard. This analysis also benefited from the organization of UniProtKB, which consists of two databases: Swiss-Prot, which is manually curated and reviewed; and TrEMBL, which is computationally generated and unreviewed. Here, we wish to identify a set of suitable databases that allow us to extend our analysis further.

We, therefore, have used the following criteria for selection of a database, firstly on technical grounds: the database must make available historical versions; contain more than just minimal amounts of textual annotation; and, be in a form which is relatively easy to obtain and parse. Within this, we have picked a set of databases of mixed maturity to obtain a reasonable sample. We chose the following five databases:

- neXtProt (Lane *et al.*, 2012)—focused solely on human proteins, neXtProt incorporates data from various sources and is built as a participative platform; the core corpus is based on human proteins from Swiss-Prot. Unlike many databases, neXtProt provides a classification system that categorizes data based on its quality into gold, silver or bronze.
- PROSITE (Sigrist *et al.*, 2013)—consists of sequence patterns, or motifs, that are conserved in protein sequences and can be used to help infer information about a sequence, such as which protein family it belongs to and its possible function. Each PROSITE entry contains a pointer to a relevant documentation entry, which provides biological information that can be inferred by the pattern.
- PRINTS (Attwood *et al.*, 2003)—a collection of sequence motifs, similar to PROSITE. However, entries in PRINTS are known as fingerprints, as they are composed of multiple motifs, unlike entries in PROSITE which contain only single motifs. All PRINTS entries are manually curated and provide cross-references to the equivalent PROSITE entries, if they exist.
- TIGRFAMs (Haft *et al.*, 2003)—provides a collection of protein families which are designed to assist with the prediction of protein function. Each TIGRFAMs entry contains a textual annotation section with additional supporting information, such as GO annotations and references to relevant Pfam and InterPro entries.
- InterPro (Hunter *et al.*, 2012)—an integrative database collating information regarding protein families, domains and functional sites from eleven member databases, including PROSITE, PRINTS and TIGRFAMs. Each InterPro entry contains a description, or abstract, which is often supplemented with references to relevant literature.

The chosen databases are summarized, along with the URL used to access each database, in Table 1.

**Table 1.** The databases chosen for our analyses, including the web address (URL) of each database

Database name	URL
UniProtKB (Swiss-Prot & TrEMBL)	http://www.uniprot.org/
InterPro	http://www.ebi.ac.uk/interpro/
neXtProt	http://www.nextprot.org
PROSITE	http://prosite.expasy.org/
PRINTS	http://130.88.97.239/PRINTS/
TIGRFAMs	http://www.jcvi.org/cgi-bin/tigrfams/

### 2.2 Data extraction and visualization

For our analysis, we need to extract sentences from the textual annotation of each database. As each of these has a different format for each of these necessitates, a custom framework was written for each, which was extended from the tool described previously (Bell *et al.*, 2013). Fortunately, the requirements for our analysis are fairly simple: we need only extract the textual annotation and basic metadata for a record (the identifier or accession number), so this process is relatively straightforward and robust to differences (or changes over time) in the database format. Sentences are intentionally extracted *verbatim* and stored in lower-case, with only database-specific formatting removed. For example, the following data from UniProtKB:

CC -!- FUNCTION: May be a transcription factor with important functions

CC in eye and nasal development.

would be transformed and stored as:

may be a transcription factor with important functions in eye and nasal

development.

This form of analysis is intentionally very simple; we performed no stemming or even stop-word analysis, with white space normalization the only change made to sentences. While this form of analysis may seem very blunt, we choose it for two reasons: it is computationally very attractive, both when parsing and searching for matches; and, most importantly, we were concerned more with correctness than recall. When a match between two databases is found, it is very likely to be a real one.

Following extraction, sentences were stored in a relational database, linked to a record identifier, database and version. Dates of records are calculated using the release version in which a record occurs, and therefore reflect an upper bound, the size of which is reflective of the release frequencies of the databases, as described previously (Bell *et al.*, 2013).

The visualization of sentence propagation uses an interactive visualization using the Highcharts library (http://www.highcharts.com/products/highcharts) driven directly from the database generated in the previous step. These visualizations provide various interactive features such as zooming, narrowing and so forth. For full details, please see (Bell, 2015).

## 3 Results

First, we introduce a number of measures that we have used to analyze reuse of textual annotation. We focus on the number of sentences within a database. It would be expected that for the sentences that occur in the database, some will occur more than once (i.e. the database is redundant) and some only once. These allow us to distinguish between the three following measures of a sentence which we reuse throughout the paper.

- Total sentences—A redundant set of all sentences in a database version.
- Unique sentences—A non-redundant set of all sentences in a database version.
- Singleton sentences—A set of sentences that occur only a single time within an entire database version.

### 3.1 Reuse within databases

Previously, we have shown that UniProtKB (i.e. Swiss-Prot and TrEMBL) reuse sentences between multiple records; in the case of TrEMBL this reuse is extreme with only 8131 unique sentences from 22 706 421 total sentences. First, we address the question of how widespread this practice of reuse is within our chosen databases. Moreover, we ask whether this is a feature of the overall size and complexity of a database.

**Fig. 1.**
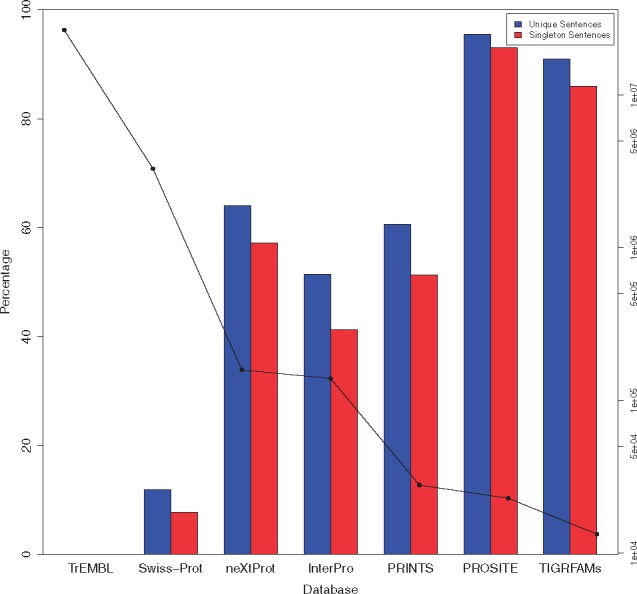
Figure showing the percentage of singleton (red/right hand column) and unique (blue/left hand column) sentences in each analyzed database. The line graph represents the total number of sentences in the database (shown on log scales). Within this graph we can broadly see that the larger the database, the more redundant its annotation (Color version of this figure is available at *Bioinformatics* online.)

To address this question, in Figure 1 we show the total number of sentences in each database; to recap, this is the number of sentences that occur in all records, whether they are duplicates or not. This is shown on a log scale as TrEMBL is much larger in size than all of the others, as shown in Table 2. In Figure 1, we also show the number of unique and singleton sentences as a percentage of the total.

**Table 2.** Table showing the total number of sentences, unique (i.e. distinct) sentences and singleton sentences contained within the latest version of each analyzed database

	Total sentences	Unique sentences	Singleton sentences	Total unique
Swiss-Prot	3 304 681	394 233	255 349	531 206
TrEMBL	26 706 421	8131	735	49 665
InterPro	139 624	71 755	57 628	100 874
neXtProt	158 929	101 822	90 875	110 607
PROSITE	22 940	21 902	21 356	29 127
PRINTS	27 987	16 953	14 356	17 858
TIGRFAMs	13 360	12 155	11 481	13 373

*Note:* Additionally, we show the total number of unique sentences over the lifetime of the entire database.

This analysis shows a number of features. First, TrEMBL is shown to be an extreme outlier; as a database, it is very large, but has the lowest number of unique sentences (8131, followed by TIGRFAMs with 12 155). Of the databases, the PROSITE database has the highest percentage of unique sentences—over 95% of sentences are unique. Broadly, this theme is also repeated in the other databases—the larger the database, the more reuse we see.

From this, we conclude that reuse of sentences is a feature of all of the databases that we have analyzed, and that this reuse is substantial in most cases.

### 3.2 Patterns of reuse within databases

Previously, we have shown that there are identifiable *patterns* of reuse within UniProtKB. We hypothesized that some of these patterns may be indicative of low quality or erroneous annotation occurring as a result of a failure to propagate changes; this was confirmed for one pattern by a close analysis of a number of examples Bell *et al.* (2013).

Having confirmed in Section 3.1 that sentence reuse is a feature of all databases that we have analyzed. We now address the question as to whether the patterns we found in UniProtKB are also present elsewhere.

We analyze the databases here for two patterns, *transient* and *missing origin*. The transient pattern is where sentences occur within an entry for only a single database release (i.e. they are removed from an entry after one iteration of the database). From this definition, it follows that it is impossible to classify a sentence as transient when it occurs only in the current version of a database, so we show these independently as *possibly transient*, although we do not consider this to be a separate pattern. A sentence follows the missing origin pattern if it initially occurs in a database entry, is later propagated to a secondary entry (or entries) and then subsequently removed from the origin entry whilst still remaining in the secondary entries. Table 3 shows the number of sentences identified in each database which follow each pattern.

**Table 3.** Table summarizing the number of sentences following the transient and missing origin propagation patterns for each database

Database name	Missing origin	Transient	Possibly transient
UniProtKB	8355	42 460	25 582
InterPro	2689	4094	1293
neXtProt	35	5148	773
PROSITE	132	2644	21
PRINTS	81	206	363
TIGRFAMs	17	563	63

*Note:* Sentences classified as possibly transient are those which appear a single time in the latest version of the database.

**Fig. 2.**
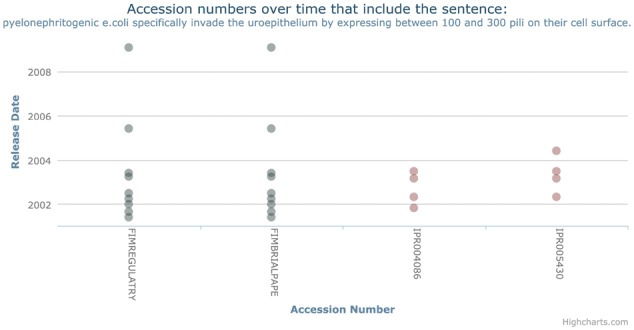
Example of sentence which follows the missing origin pattern. Here, the sentence originates in InterPro entry IPR004086 before later appearing in entry IPR005430. It remains in this entry even when then sentence is removed from IPR004086. Interestingly, we note that the sentence occurs in PRINTS both before and after it exists in InterPro

From these results, we note that all of the databases show incidences of the patterns that we have previously identified. Of the databases, PRINTS and TIGRFAMs have the lowest level of all of these patterns. This is consistent with Figure 1—as these patterns are a feature of a unique sentence, they are upper-bounded by the uniqueness, and likely to be affected by the level of reuse within the databases. To be classified, a sentence only needs to exhibit a pattern in a single entry. A clear example of this is shown in Figure 2 which shows an example of the missing origin pattern. This sentence (‘pyelonephritogenic e.coli specifically invade the uroepithelium by expressing between 100 and 300 pili on their cell surface’) initially appears in InterPro entry IPR004086 in 2001 and later appears in InterPro entry IPR005430 approximately a year later. However, the sentence is removed from IPR004086 (the origin) in 2003 while still remaining in the secondary entry IPR005430 for another release.

We have chosen this example, because it clearly represents an error in the database, albeit a minor typographical one; namely the presence of a space between the species and genus in ‘E. coli’ (This modification seems to reflect a change in the underlying XML representation as taxonomic markup was removed at the same time. Our analysis explicitly excludes markup early in the pipeline; we note, however, that were it included, the missing origin pattern would also have detected the lack of percolation of markup changes.). Obviously this form of the error is unlikely to cause major challenges for human consumption of the database annotation, but could cause issues for computational use.

In this section, therefore, we have demonstrated that the patterns of reuse that we have previously seen in UniProtKB also occur in other databases and in some cases, at reasonably high levels. In general, these patterns occur more in databases with more redundancy.

### 3.3 Reuse between databases

One common worry about knowledge in biology is that it is circular, as the knowledge is reused and percolated through the biological database ecosystem. If this is true, then we should be able to detect this supposed reuse, for instance by sentence reuse *between* databases. In fact Figure 2, in addition to showing a missing original, also shows an example of reuse between databases; a sentence which appears first in PRINTS and, then, later reappears in InterPro.

To address this question more systematically, we have looked for identical sentences that occur between any of the databases in our collection, the results of which are shown in Table 4.

**Table 4.** Table summarizing the distribution of all unique sentences shared between the analyzed databases

Database combination	Total sentences
UniProtKB	526 435
neXtProt; UniProtKB	83 868
InterPro	82 968
neXtProt	26 539
PROSITE	23 182
PRINTS	10 064
TIGRFAMs	9661
InterPro; PRINTS	7751
InterPro; PROSITE	5790
InterPro; TIGRFAMs	3681
InterPro; UniProtKB	435
InterPro; neXtProt; UniProtKB	151
PROSITE; UniProtKB	71
InterPro; PROSITE; UniProtKB	26
neXtProt; PROSITE; UniProtKB	20
InterPro; PRINTS; UniProtKB	20
InterPro; neXtProt; PROSITE; UniProtKB	19
InterPro; PRINTS; PROSITE	14
TIGRFAMs; UniProtKB	14
InterPro; TIGRFAMs; UniProtKB	9
InterPro; neXtProt; PRINTS; UniProtKB	4
neXtProt; TIGRFAMs; UniProtKB	3
InterPro; neXtProt; TIGRFAMs; UniProtKB	2
InterPro; TIGRFAMs; PROSITE	2
InterPro; neXtProt; PRINTS; PROSITE; UniProtKB	1
InterPro; PRINTS; PROSITE; UniProtKB	1
PRINTS; UniProtKB	1
PRINTS; PROSITE	1
PRINTS; TIGRFAMs	1

These results show that there is substantial reuse of sentences in two key cases. Firstly, there is a very high-level of reuse between UniProtKB and neXtProt. This is expected as neXtProt explicitly depends on UniProtKB—in this case, perhaps, it is more surprising that a significant proportion of neXtProt is unique to it (around 25% of the total sentences in neXtProt). A second case is shown between the InterPro database and PRINTS, PROSITE and TIGRFAMs. This is to be expected as InterPro is a federated database, explicitly depending on the other three databases. We do also see reuse between other databases, although this occurs at a fairly low-level, compared to the total number of sentences. There is one sentence which occurs in all five of the databases which is *‘visual pigments are the light-absorbing molecules that mediate vision.’*

From this we conclude that knowledge does percolate between different databases and that it is possible to detect this by using whole sentence analysis. However, in the majority of cases where identical sentences are found in large numbers between databases, occur as a result of a formal relationship between the two—for instance, between UniProtKB and neXtProt.

### 3.4 Patterns between databases

As we have shown previously, and in this paper, it is possible to detect patterns of reuse within databases, and that in some cases these patterns appear to be related to errors of percolation. Further, we know that, in some cases, sentence percolation can also be seen between databases. This raises the question as to whether we could detect patterns that occur between databases.

While we do have algorithms for pattern detection within a database, the same process turns out to be considerably harder between databases, mostly because of the lack of co-ordinated release dates. If a database record contains a sentence which is removed between two releases, for example, should it be considered present only till the first release, or till just before the second? When comparing two databases, these problems are significant, as the second database may have undergone several releases subsequently.

**Fig. 3.**
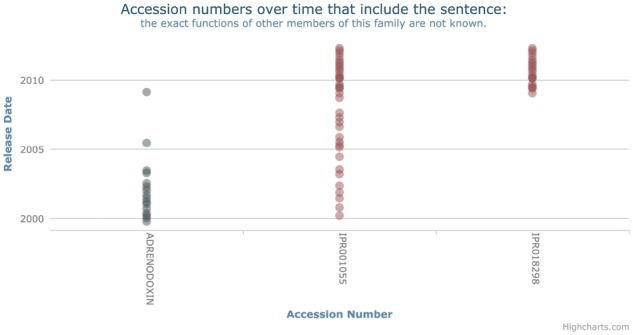
An example of a sentence in InterPro which does not follow any propagation pattern. However, if you also consider PRINTS, and the sentence was copied from PRINTS into InterPro, then the sentence technically follows the missing origin pattern. This would have significant impact on the potential correctness of sentences in all databases

As a result of these issues, we have not yet been able to address the question of pattern occurrence systematically between all databases. However, we have been able to find specific examples by inspection. We show one of these in Figure 3. In this case, a sentence appears first in PRINTS (in around 1999), and then later in 2000 appears, presumably by percolation, in InterPro first in one record (IRP001055) and then later in 2008 in another (IPR018298). Around the same time, it disappears from the original entry.

Interestingly, it is not possible to detect the occurrence of this pattern just by considering a single database. In PRINTS, the sentence occurs at one point, then stops later. In InterPro, it continues to occur in all records that it has percolated to. It is only by considering the removal from PRINTS, and the continued occurrence in InterPro that we see an instance of the missing origin pattern. This does suggest that cross-database comparisons may reveal more knowledge than the consideration of a single database.

Of course, inspection of this form is not a scalable mechanism for detecting instances of these patterns; however, without co-ordinated release dates, automation is hard to achieve. Despite this, our initial analysis indicates that there are examples of reuse patterns that are detectable between different databases and, indeed, patterns that are only detectable by considering multiple databases.

## 4 Discussion

It is often said that knowledge in bioinformatics is frequently reused, and moves through the database infrastructure. In this paper, we have attempted to investigate this in as direct a manner as possible, by looking for exact reuse of sentences between different databases. As a result of this we have found that, indeed, reuse of knowledge within bioinformatics databases is extremely common. In the most extreme case for a manually curated database, UniProtKB/Swiss-Prot, some 91% of the sentences occur more than once. For TrEMBL, this is even more skewed where unique sentences number less than 1% of the total sentences. To our knowledge, this demonstrates the first system attempt to detect, investigate and record the impact of this knowledge flow.

This reuse is, perhaps, a reflection of evolution of these databases. By itself, it is not necessarily a problem, however, it is a cause for concern. It does mean that the annotation is heavily *denormalised*—that is, what is effectively the same data is stored multiple times within a single database. This presents significant difficulties during updates; if a duplicated piece of knowledge needs to be updated with respect to a single record, then perhaps it also needs to be updated with respect to another.

We have previously shown that it is possible to detect errors or low-quality annotation, resulting from this denormalization, by looking for specific patterns of provenance in the database (Bell *et al.*, 2013). In this paper, we have shown that two of these patterns, the missing origin and transient patterns, are also present within other databases; in the case of the missing origin pattern, this is clearly in the systematic representation of species names. Further, we have shown that reuse also occurs between databases although, in general, this happens at a fairly low-level. Even here, though, it is possible to detect patterns between databases where they are not detectable from a single database.

The work described here shows the value and importance of historical records, and that this value is also relevant to the present. We have previously made extensive use of historical records when looking at trends in database word usage (Bell *et al.*, 2012), as have others to determine when a database might be complete (Baumgartner *et al.*, 2007), or to assay the accuracy of predictive tools (Gross *et al.*, 2009). These analyses have dealt with both the structured (GO) and unstructured (comments) components of annotation. This demonstrates that an accurate record of the past is useful to increase our understanding of the current state of the annotation; truly, understanding the past is useful to correcting the errors of the present.

However, there are important limitations. In our previous work, we were more able to investigate some of the instances of annotation patterns in detail, and demonstrate that they were actually errors. In this work, we were greatly aided by the existence of UniSave (Leinonen *et al.*, 2006) which allowed us to rapidly and efficiently investigate the historical record. UniProtKB is unusual in providing this form of tool however.

We can compare this to Wikipedia which includes a more complete feature set with respect to versioning than any of the bioinformatics databases that we have analyzed (with UniProtKB coming a notable second best). It does demonstrate that it is possible to store a fine-grained full version history for even a very large knowledge base. That it is searchable using the current schema is an added bonus and would greatly help this form of analysis; in fact, Wikipedia has been used as the basis for analysis of historical resources (Viégas *et al.*, 2004). Interestingly, in the last few years, PFAM has moved toward using Wikipedia as the main mechanism for maintaining their textual annotation (Punta *et al.*, 2012); while we do not believe this was the original intention, from the point-of-view of this analysis, this move should increase the quality of the historical data available.

The second critical limitation of our work is that we are not looking directly at provenance but inferring from the occurrence of identical sentences. In our work, we have erred on the side of caution by using direct string matching; this is a very useful tool for two reasons: firstly, it is computationally very simple, and extremely scalable and secondly it gives a high-level of confidence that a match does actually demonstrate knowledge flow. It is, however, also a very blunt tool, and we are likely to be missing many examples of information flow. Small changes to sentences, including grammatical or textual corrections, will break the provenance trail; indeed, we have a direct example of this happening. Moreover, when tracking provenance between databases, we suspect that database authors have a positive incentive to alter text to avoid issues of copyright or plagiarism, inadvertently making the provenance even harder to track.

A third issue with tracking provenance is the difficulty of dating individual sentences. Databases are normally developed continuously, but only released periodically, and it is the releases that we have tracked. These problems are exacerbated between databases, as the release date is the only information we have to infer the direction of the travel of knowledge. Taken together, these limitations mean that our understanding of provenance is heuristic and may be wrong. In short, our ability to exploit this knowledge is curtailed by the limited provenance information that is stored.

There are practical steps that current database provider could take which could increase our knowledge. Most software engineering projects make use of version control, which can store practically unlimited provenance of source code. Wikipedia (and, therefore, also PFAM) use the same technology for their textual annotation. This may provide a simple solution for many biological databases; it would, at least, address the requirement for fine-grained date information. Alternatively, a more formal model of provenance (such as PROV (Missier *et al.*, 2013)) might be used, which could potentially provide a more fine-grained dataset describing the relationships between sentences explicitly. This is also likely to be necessary for larger databases such as TrEMBL, which may be less suited to version control systems because of their size, automatic generation and relatively low levels of textual annotation per entry.

Despite these limitations, we have shown that knowledge flows between databases even when there is not a formal link between them. While this raises the spectre that some of the knowledge in these databases may be circular, we have also shown that it is possible to detect patterns which may lead to mechanisms of error detection, which should increase the quality of knowledge in biology.
